# Associations between Endothelial Lipase, High-Density Lipoprotein, and Endothelial Function Differ in Healthy Volunteers and Metabolic Syndrome Patients

**DOI:** 10.3390/ijms24032073

**Published:** 2023-01-20

**Authors:** Iva Klobučar, Julia T. Stadler, Lucija Klobučar, Margarete Lechleitner, Matias Trbušić, Gudrun Pregartner, Andrea Berghold, Hansjörg Habisch, Tobias Madl, Gunther Marsche, Saša Frank, Vesna Degoricija

**Affiliations:** 1 Department of Cardiology, Sisters of Charity University Hospital Centre, 10000 Zagreb, Croatia; 2 Otto Loewi Research Center, Division of Pharmacology, Medical University of Graz, 8010 Graz, Austria; 3 Department of Medicine, University Hospital Centre Osijek, 31000 Osijek, Croatia; 4 Gottfried Schatz Research Center, Molecular Biology and Biochemistry, Medical University of Graz, 8010 Graz, Austria; 5 School of Medicine, University of Zagreb, 10000 Zagreb, Croatia; 6 Institute for Medical Informatics, Statistics und Documentation, Medical University of Graz, 8036 Graz, Austria; 7 BioTechMed-Graz, 8010 Graz, Austria; 8 Department of Medicine, Sisters of Charity University Hospital Centre, 10000 Zagreb, Croatia

**Keywords:** high-density lipoprotein, endothelial lipase, flow-mediated dilation, NMR spectroscopy, metabolic syndrome

## Abstract

Metabolic syndrome (MS) is characterized by endothelial- and high-density lipoprotein (HDL) dysfunction and increased endothelial lipase (EL) serum levels. We examined the associations between EL serum levels, HDL (serum levels, lipid content, and function), and endothelial function in healthy volunteers (HV) and MS patients. Flow-mediated dilation (FMD), nitroglycerin-mediated dilation (NMD), serum levels of HDL subclasses (measured by nuclear magnetic resonance (NMR) spectroscopy), and EL serum levels differed significantly between HV and MS patients. The serum levels of triglycerides in large HDL particles were significantly positively correlated with FMD and NMD in HV, but not in MS patients. Cholesterol (C) and phospholipid (PL) contents of large HDL particles, calculated as HDL1-C/HDL1-apoA-I and HDL1-PL/HDL1-apoA-I, respectively, were significantly negatively correlated with FMD in HV, but not in MS patients. Cholesterol efflux capacity and arylesterase activity of HDL, as well as EL, were correlated with neither FMD nor NMD. EL was significantly negatively correlated with HDL-PL/HDL-apoA-I in HV, but not in MS patients, and with serum levels of small dense HDL containing apolipoprotein A-II in MS patients, but not in HV. We conclude that MS modulates the association between HDL and endothelial function, as well as between EL and HDL. HDL cholesterol efflux capacity and arylesterase activity, as well as EL serum levels, are not associated with endothelial function in HV or MS patients.

## 1. Introduction

Ischemic heart disease has been the leading cause of death in adults in high- and middle-income countries worldwide within the last decades [1]. Therefore, the pathophysiology of atherosclerosis and its preceding stages remains a focus of research.

The vascular endothelium maintains a normal vascular tone through an interplay between endothelium-derived relaxing and contracting factors [2]. Endothelial dysfunction is considered an early stage of atherosclerosis that can be detected even before the occurrence of structural changes to the vessel wall that are visible using ultrasonography or angiography [3]. Endothelial dysfunction is characterized by decreased nitric oxide (NO) availability. Endothelial dysfunction and impaired smooth-muscle cell NO reactivity, together with a predominance of reactive oxygen species and increased vascular inflammation, promote atherosclerosis [4]. Endothelial (dys)function may be evaluated in vivo by measuring and comparing flow- and nitroglycerin-mediated dilation (FMD and NMD) of the brachial artery [5].

High-density lipoprotein (HDL) represents a heterogeneous mixture of nanoparticles that differ in size, lipid, and protein composition, as well as function [6]. In addition to its role in mediating reverse cholesterol transport, HDL contributes to the maintenance of normal endothelial function by the promotion of endothelial NO production, antioxidative and anti-inflammatory effects, as well as by the regulation of the endothelial cell thrombotic activation [6,7,8].

Endothelial lipase (EL) is an enzyme with substantial phospholipase and less pronounced triglyceride lipase activity, expressed primarily by vascular endothelial cells. EL is a negative regulator of HDL plasma levels [9,10,11,12] and an important modulator of HDL composition and function [12,13,14,15,16,17].

Metabolic syndrome (MS) is a pathophysiological condition characterized by central obesity, dyslipidemia, arterial hypertension, and hyperglycemia [18]. It has been shown that each of these risk factors promote the occurrence of endothelial dysfunction [19]. Moreover, all MS constituents are positively associated with EL plasma levels [20], and the ability of HDL to stimulate endothelial NO production has been found to be impaired in MS patients [21]. However, there is in vitro and ex vivo evidence that HDL modified by EL exhibits an increased NO-inducing and vasorelaxant activity [14]. Based on these facts, we hypothesized that EL, by its positive effect on the NO-inducing activity of HDL, positively affects the endothelial function in healthy subjects, whereas an overall impairment of the HDL and endothelial function offsets this effect of EL in MS patients.

In contrast to our previous studies addressing the impact of EL on the HDL composition and function in vitro and ex vivo [14,15,16,17], as well as the association of EL with HDL serum levels, composition, and function in healthy subjects and acute heart failure patients [17,22], we examined here for the first time the associations between EL and HDL (serum levels, lipid content, and function), as well as between EL, HDL, and the in vivo measured vascular reactivity of a brachial artery in healthy volunteers (HV) and MS patients.

## 2. Results

### 2.1. Demographics, Clinical Characteristics, and Medication

A total of 130 participants were enrolled in the study, 65 HV and 65 MS patients. The participants’ demographic and clinical characteristics are presented in Table 1. Although not specifically matched, the groups were systematically balanced regarding age and sex. Furthermore, the HV and MS groups did not differ significantly regarding age and sex, body height, smoking status, and presence of a regular menstrual cycle in women. MS patients had significantly higher body weight and thus had a higher body mass index (BMI), a larger waist circumference, and lower levels of physical activity per week compared to the HV group. Arterial hypertension and diabetes mellitus type 2 were the most common chronic diseases present in the MS groups, affecting 92.3% and 41.5%, respectively. Regarding medication, 23 (35.4%) MS patients were treated with each statin, metformin, and beta-blocker, and 25 (38.5%) with diuretics. None of these were used in the HV group.

### 2.2. Standard Laboratory Data

Compared to HV, MS patients had significantly higher EL and triglyceride levels, as well as significantly lower high-density lipoprotein cholesterol (HDL-C) serum levels. While serum levels of glucose, protein, C-reactive protein (CRP), interleukin-6 (IL-6), alanine aminotransferase (ALT), gamma-glutamyl transpeptidase (GGT), creatine kinase (CK), urea, and urate were significantly higher, serum levels of bilirubin, sodium, and chloride were significantly lower in MS patients, compared to HV. Total cholesterol, low-density lipoprotein cholesterol (LDL-C), albumin, aspartate aminotransferase (AST), alkaline phosphatase (AP), and lactate dehydrogenase (LDH), as well as creatinine, estimated glomerular filtration rate (eGFR), and potassium, were not significantly different between the groups (Table 2).

### 2.3. HDL Subclasses, Lipid Content, and Function

Serum levels of total and subclasses 1–4 of HDL-C, HDL-phospholipids (HDL-PL) and HDL-apolipoprotein A-I (HDL-apoA-I) were significantly lower in MS patients compared to HV. In contrast, serum levels of total and subclasses 2–4 of HDL-triglycerides (HDL-TG) were significantly higher in MS patients compared to HV (Table 3). While serum levels of total HDL-apolipoprotein A-II (HDL-apoA-II), as well as of subclass 1, were significantly lower in MS patients compared to HV; the subclasses 2–4 of HDL-apoA-II were similar in both groups (Table 3).

We used HDL-apoA-I as a rough estimate of HDL particle number and calculated ratios of HDL lipids to HDL-apoA-I to estimate the lipid content of HDL subclasses. While the cholesterol content of total HDL and subclasses 2–4, as well as phospholipid content of total HDL and subclass 4, were significantly lower, the triglyceride contents of total HDL and subclasses 1–4 were significantly higher in MS patients compared to HV (Table 4).

Apolipoprotein B-depleted serum was used as an HDL surrogate to determine HDL function. Both arylesterase activity of HDL-associated paraoxonase 1 (AE activity) and HDL cholesterol efflux capacity were significantly lower in MS patients compared to HV. However, the difference between the groups was nullified when the AE activity and CEC were normalized to HDL-apoA-I, indicating that the differences in HDL abundance rather than in HDL function were responsible for the observed differences between the groups (Table 5).

### 2.4. Brachial Artery Function

**Figure 1:**
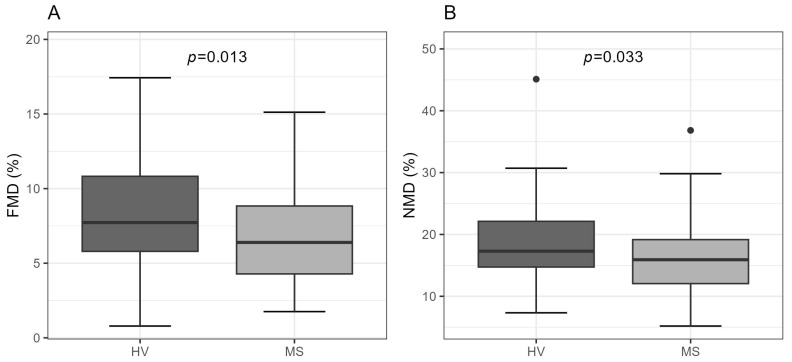
Boxplots of (**A**) FMD and (**B**) NMD in HV vs. MS patients. Data are presented as boxplots with median and range. Differences between the groups were tested with a t-test. HV, healthy volunteer; FMD, flow-mediated dilation; MS, metabolic syndrome patient; NMD, nitroglycerin-mediated dilation.

Ultrasonographic measurements of the brachial artery function revealed significantly lower FMD (HV: 8.1 ± 3.5%; MS: 6.7 ± 3.1%; *p* = 0.013) and NMD (HV: 18.4 ± 6.6 %; MS: 16.0 ± 5.7%; *p* = 0.033) values, as well as a longer time to maximal brachial artery dilation during the FMD measurement (HV: 50.0 (41.0, 67.0) s; MS: 75.0 (56.0, 99.0) s; *p* < 0.001) in MS patients compared to HV (Figure 1 and Table S1).

In contrast, the time to maximal brachial artery dilation after nitroglycerin application during the NMD measurement was similar in both groups (Table S1). Additionally, in the MS group, FMD and NMD were similar in the patients with or without chronic treatment with statins (FMD: *p* = 0.945; NMD: *p* = 0.330), metformin (FMD: *p* = 0.118; NMD: *p* = 0.476), beta-blockers (FMD: *p* = 0.794; NMD: *p* = 0.442), or diuretics (FMD: *p* = 0.914; NMD: *p* = 0.559). Complete results of the ultrasonographic measurements of the brachial artery function are presented in Table S1.

### 2.5. Correlation Analyses of HDL Subclasses, Lipid Content, and Function with FMD and NMD

**Figure 2:**
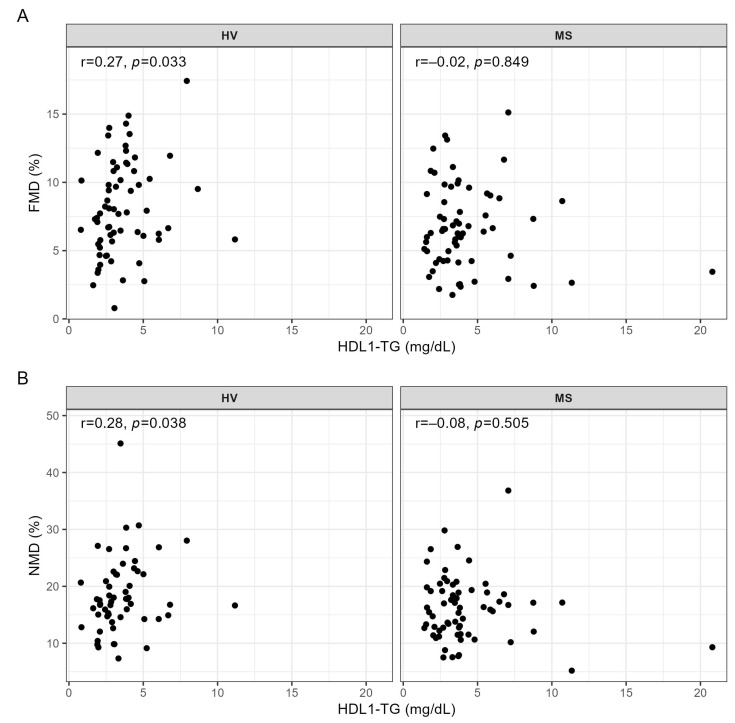
Correlations of HDL1-TG with (**A**) FMD and (**B**) NMD in HV and MS patients. Correlations were quantified using Spearman’s correlation coefficient. dL, deciliter; HDL, high-density lipoprotein; HV, healthy volunteer; FMD, flow-mediated dilation; mg, milligram; MS, metabolic syndrome patient; NMD, nitroglycerin-mediated dilation; r, Spearman’s correlation coefficient; TG, triglyceride.

Regarding the associations of serum levels of the HDL subclasses with metrics of the vessel function, we observed a significant positive correlation of HDL1-TG serum levels with FMD and NMD in HV, but not in MS patients (Figure 2A,B). 

**Figure 3:**
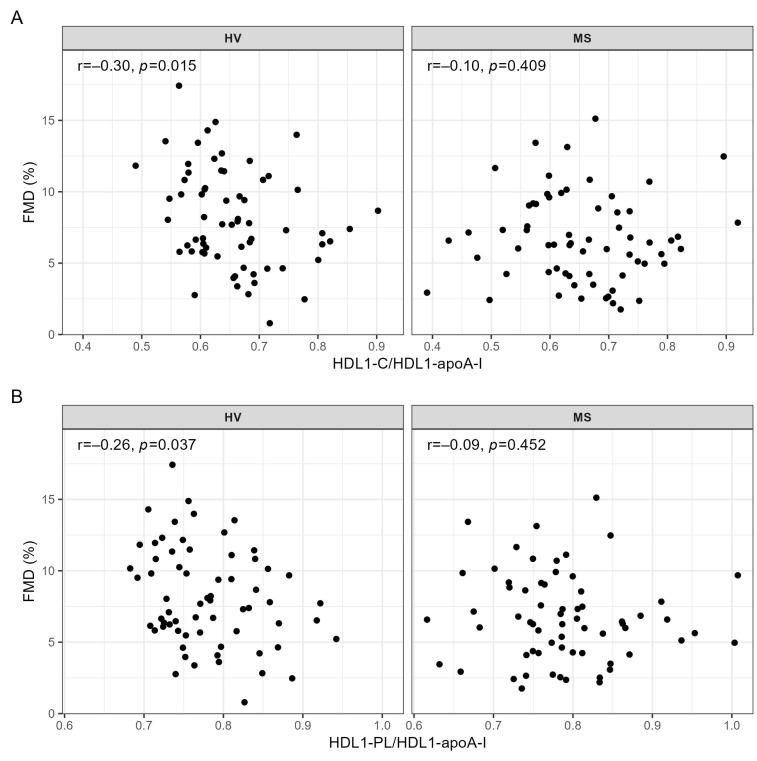
Correlations of (**A**) HDL1-C/HDL1-apoA-I and (**B**) HDL1-PL/HDL1-apoA-I with FMD in HV and MS patients. Correlations were quantified using Spearman’s correlation coefficient. ApoA-I, apolipoprotein A-I; C, cholesterol; HDL, high-density lipoprotein; HV, healthy volunteer; FMD, flow-mediated dilation; MS, metabolic syndrome patient; PL, phospholipid; r, Spearman’s correlation coefficient.

There were no further significant correlations between other HDL subclasses and the metrics of vessel function (Table S2). However, when testing the associations between the ratios indicating the lipid content of HDL particles and vessel function, we found a significant negative correlation of HDL1-C/HDL1-apoA-I and HDL1-PL/HDL1-apoA-I with FMD in HV, but again not in MS patients (Figure 3A,B and Table S3). 

Neither HDL1-C/HDL1-apoA-I nor HDL1-PL/HDL1-apoA-I were significantly correlated with NMD (Table S3). No other ratios indicating the lipid content of HDL particles or the metrics of HDL function (AE activity and CEC) were significantly correlated with FMD or NMD (Tables S3 and S4). 

### 2.6. Correlation Analyses of EL with Metrics of Vessel Function, HDL Subclasses, Lipid Content, and Function

**Figure 4:**
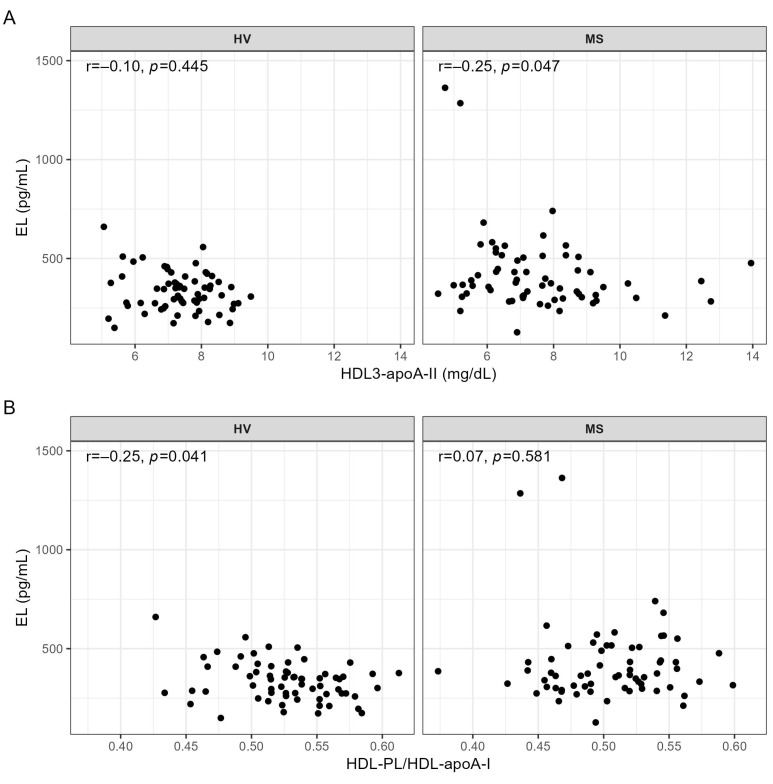
Correlations of EL with (**A**) HDL3-apoA-II and with (**B**) HDL-PL/HDL-apoA-I in HV and MS patients. Correlations were quantified using Spearman’s correlation coefficient. ApoA-I, apolipoprotein A-I; apoA-II, apolipoprotein A-II; EL, endothelial lipase; HDL, high-density lipoprotein; HV, healthy volunteer; MS, metabolic syndrome patient; PL, phospholipid; r, Spearman’s correlation coefficient.

EL serum levels were not significantly correlated with FMD (HV: r = 0.22, *p* = 0.084; MS: r = 0.19, *p* = 0.139) or NMD (HV: r = 0.20, *p* = 0.133; MS: r = 0.07, *p* = 0.561). We only found one significant association between the EL serum levels and the HDL subclasses, namely a significant negative correlation with HDL3-apoA-II in MS patients, but not in HV (Figure 4A and Table S5). Regarding the association of EL with the lipid content of HDL particles, we observed a significant negative correlation of EL with HDL-PL/HDL-apoA-I in HV, but not in MS patients (Figure 4B and Table S6).

There were no significant correlations of EL with other ratios indicating the lipid content of the HDL particles or with metrics of HDL function (Tables S6 and S7). 

## 3. Discussion

In the present study, we provide evidence that associations between EL, HDL, and endothelial function are different in HV and MS patients.

In vitro experiments in primary endothelial cells, as well as in animal models, have established the endothelial function of HDL, exemplified by the profound capacity of HDL to increase the endothelial NO availability and promote vasodilation [8,23,24,25]. Ample evidence for the NO-inducing and vasodilating capacity of HDL in humans came from a study in which intravenous injection of reconstituted HDL into hypercholesterolemic patients increased acetylcholine-induced vasodilation by increasing the NO bioavailability [26]. In line with this, several studies reported a positive association between HDL-C levels and FMD [27,28,29,30,31]. However, several other studies showed either no association or even found a negative association between HDL-C and FMD [32,33,34,35,36]. 

In the present study, we found a significant positive association of HDL1-TG, which represents the serum levels of triglycerides in large buoyant HDL particles, with both FMD and NMD in HV, but not in MS patients. The fact that the strength of the association of HDL1-TG with FMD was similar to that with NMD (Figure 2) suggests that the serum levels of HDL1-TG are associated with the sensitivity and responsiveness of vascular smooth muscle cells to NO rather than with the endothelial function and NO production. The lack of association of HDL1-TG with FMD and NMD in MS patients (Figure 2), despite similar levels of HDL1-TG in both groups (Table 3), probably reflects a detrimental effect of MS on the vascular function, illustrated by significantly lower FMD and NMD in MS patients compared to HV (Figure 1). A mechanistic link between HDL1-TG and the responsiveness of the vascular smooth muscle cells to NO is presently not clear and should be addressed in future research. 

We used HDL-apoA-I as a rough estimate of the HDL particle number and calculated ratios of HDL lipids to HDL-apoA-I to estimate the lipid content of the HDL subclasses. We show here for the first time that the cholesterol and phospholipid contents of large HDL particles, calculated as HDL1-C/HDL1-apoA-I and HDL1-PL/HDL1-apoA-I, respectively, are significantly negatively correlated with the endothelial function in HV, but not in MS patients (Figure 3). It is well-established that by binding to the endothelial scavenger receptor B-type I (SR-BI), HDL promotes cholesterol efflux and activates eNOS activity and NO production by the vascular endothelium [23,37,38]. Furthermore, previous studies have shown that the extent of the cholesterol and phospholipid load per HDL particle critically affects the interaction of HDL with SR-BI and the efficacy of cholesterol efflux [13,15,39,40]. Accordingly, the observed negative associations of FMD with HDL1-C/HDL1-apoA-I and HDL1-PL/HDL1-apoA-I likely reflect the diminishing effect of physiological HDL enrichment with cholesterol and phospholipids on the interaction of HDL with the SR-BI/eNOS/NO pathway. Additionally, since HDL-PL, as quantified by NMR spectroscopy, reflects HDL phosphatidylcholine as well as lysophosphatidylcholine, the higher HDL1-PL/HDL1-apoA-I ratio might reflect an enrichment of HDL with lysophosphatidylcholine, which by inducing oxidative stress, diminishes the endothelial NO availability and attenuates the endothelium-dependent vasorelaxation [41]. 

EL is an established determinant of HDL serum levels in mice and humans [10,11,12,42]. However, previous studies reported a negative correlation between EL and HDL serum levels in patients with cardiovascular diseases, but not in those without [17,22,43,44]. In line, in the present study, EL serum levels were not correlated with any of the parameters of HDL bioavailability measured by NMR spectroscopy in HV, but were negatively correlated with small dense HDL which contain apoA-II (HDL3-apoA-II) in MS patients (Figure 4A). This finding is novel and might be related to the negative impact of HDL-associated apoA-II on the ability of EL to influence the metabolism of HDL, as observed in double transgenic human apoA-I/apoA-II mice overexpressing human EL [45]. To clarify how MS pathophysiology, which promotes a positive association of EL with BMI, IL-6, and CRP (Table S8), promotes the negative association between EL and HDL3-apoA-II in humans needs further investigation. 

In agreement with a pronounced phospholipase activity of EL and a high affinity of EL for HDL phospholipids [9,15,16,17,46,47], we observed a negative correlation between EL and HDL phospholipid content (HDL-PL/HDL-apoA-I) in HV, but not in MS patients (Figure 4B). It is conceivable that in MS, despite higher EL serum levels compared to HV (Table 2), the decreased activity of lipoprotein lipase, an enzyme involved in the biogenesis of HDL [48], as well as the degradation of HDL phospholipids by upregulated other serum phospholipases [49,50], or adiposity, known to affect HDL size, composition, and subclass distribution [51], mask the impact of EL on the phospholipid content of HDL. 

Although in vitro EL-modified HDL exhibits an increased NO-inducing and vasorelaxant activity [14], EL serum levels were not correlated with FMD neither in HV nor MS patients. This implies that the EL modification of HDL in the human circulation does not result in an improved quality of HDL or, alternatively, that a complex (patho)physiological environment, with complex neurohormonal regulatory mechanisms and a number of confounders in the circulation and the vessel wall, mask the effect of EL and the beneficial endothelial action of EL-modified HDL. 

This study has several limitations. Since the applied NMR methodology does not provide concentrations of HDL particles, we used HDL-apoA-I, which is a rough estimate of the HDL particle concentration, for the calculation of the ratios indicative of the lipid content of HDL particles. Considering that EL serum levels in the present study were determined in pre-heparin serum, the associations of post-heparin EL levels with HDL or FMD could be different from those observed. However, previous studies showed that pre- and post-heparin EL plasma levels are highly correlated or similar [20,43]. Of note, EL mass does not necessarily reflect EL enzyme activity, which is known to be affected by genetic EL polymorphisms, as well as endogenous inhibitors, such as angiopoetin-like protein 3, protein convertases, or apoA-II [11,12,42,45,52,53,54]. Therefore, the association of EL activity with the HDL subclasses or FMD might be different from what we found for the EL mass in the present study. Indeed, in contrast to no observed association between EL serum levels and HDL-C in the present study, a previous study found a significant association of EL activity with HDL-C in both healthy controls and MS patients [48]. 

Based on our results, we conclude that the complex MS pathophysiology disrupts the negative associations of HDL cholesterol and phospholipid content with FMD, as well as of HDL phospholipid content with EL observed in HV, but promotes the association of EL with small dense HDL, which contains apoA-II. Despite not observing a direct association of EL with endothelial function, the EL-mediated depletion of HDL phospholipids might be a driving factor for the negative association of the HDL phospholipid content with FMD in HV. If so, blocking EL with monoclonal antibodies, an approach which, in humans, increases HDL-C and HDL particle numbers, as well as HDL cholesterol efflux and anti-inflammatory activity [55], would conceivably increase phospholipid content but decrease the endothelial function of HDL. However, intervention studies are needed to examine whether manipulation of EL serum levels or activity translates into clinical benefits for patients with MS or other pathologies associated with impaired HDL and endothelial function.

## 4. Materials and Methods

### 4.1. Study Design and Participants

We present the results of an observational, cross-sectional study that included a total of 130 individuals aged 45 to 65 years; 65 HV and 65 MS patients. MS was defined by five internationally unified criteria [56] and diagnosed if at least three criteria were met. Waist circumference thresholds of ≥102 cm in men and ≥88 cm in women were considered appropriate for the study population. The presence of any chronic disease was an exclusion criterion for HV, while the history of myocardial infarction, cardiomyopathy, severe renal insufficiency (eGFR ≤29 mL/min/1.73 m^2^), liver cirrhosis (Child Pugh stages B and C), and malignant and autoimmune diseases were exclusion factors for MS patients. Any kind of recent acute infectious or inflammatory condition and hypersensitivity associated with the use of glyceryl trinitrate were exclusion criteria for both groups. All study participants were asked not to consume food and caffeine 8 to 12 h prior, to refrain from smoking and physical exercise, as well as to suspend any vasoactive medications and vitamin preparations 24 h prior to the study visit. The study was approved by the local ethics committees of the Sisters of Charity University Hospital Centre, Zagreb, Croatia (EP 13125/17-4), the University of Zagreb School of Medicine, Croatia, and the Medical University of Graz, Austria (31-532 ex 18/19). Prior to enrolment in the study, all participants signed informed consent. The study was performed in accordance with the principles of Good Clinical Practice Guidelines and the Declaration of Helsinki [57].

### 4.2. Laboratory Procedures

A sample of venous blood was obtained from each individual after 8 to 12 h of fasting and within 15 min before the assessment of vascular function. The blood was collected in four 9 mL tubes of a VACUETTE^®^ Z Serum Clot Activator (Greiner Bio-One GmbH, Kremsmuenster, Austria). The tubes were incubated for 30 min at room temperature and subsequently centrifuged at 1800× *g* for 10 min at 4 °C. Total cholesterol, HDL-C, triglycerides, and CRP were measured by using the Cobas c system (Roche Diagnostics, Hitachi, Tokyo, Japan) and LDL-C was calculated using Friedewald’s formula [58]. Other routine laboratory analyses, including serum glucose, total protein, albumin, bilirubin, ALT, AST, AP, GGT, LDH, CK, creatinine, urea, urate, sodium, potassium, and chloride, were measured using Cobas 8000 (Roche Diagnostics, Hitachi, Tokyo, Japan). eGFR was calculated according to Levey et al. [59]. EL serum levels were measured using a Human EL-Assay Kit (TaKaRa, Takara Bio Europe S.A.S., Saint-Germain-en-Laye, France), as described previously [17]. IL-6 was quantified by electro-chemiluminescence immunoassay using the Cobas e801 system (Roche Diagnostics, Hitachi, Tokyo, Japan). 

### 4.3. Lipoprotein Profiling Using Nuclear Magnetic Resonance (NMR) Spectroscopy

Serum levels of total HDL-C, HDL-TG, HDL-PL, HDL-apoA-I, and HDL-apoAII, as well as of their 4 size/density subclasses (HDL1: 1.063–1.100 kg/L; HDL2: 1.100–1.112 kg/L; HDL3: 1.112–1.125 kg/L; HDL4: 1.125–1.210 kg/L), were measured on a Bruker 600 MHz Avance Neo NMR spectrometer using the Bruker IVDr lipoprotein subclass analysis protocol, as described [17,60]. Briefly, serum samples were thawed, and 330 µL of each sample was mixed with 330 µL of Bruker serum buffer (Bruker, Rheinstetten, Germany). The samples were mixed gently and 600 µL of the mixed sample was transferred into a 5 mm SampleJet rack tube (Bruker). Proton spectra were obtained at a constant temperature of 310 K using a standard Nuclear Overhauser Effect Spectroscopy (NOESY) pulse sequence (Bruker: noesygppr1d), a Carr–Purcell–Meiboom–Gill (CPMG) pulse sequence with presaturation during the relaxation delay (Bruker: cpmgpr1d) to achieve water suppression, and a standard 2D J-resolved (JRES) pulse sequence (Bruker: jresgpprqf). Data analysis was carried out using the Bruker IVDr LIpoprotein Subclass Analysis (B.I.LISA^TM^, Bruker Biospin, Rheinstetten, Germany).

### 4.4. Metrics of HDL Function

Metrics of HDL function were measured using apoB-depleted serum generated as a supernatant, following incubation of 100 µL of serum with 40 μL of polyethylene glycol at room temperature for 20 min and centrifugation at 10 000 rpm and 4 °C for 20 min. AE activity was assessed using a photometric assay with phenylacetate as a substrate, as described previously [17]. In brief, apoB-depleted serum was diluted 10-fold and 1.5 μL was added to 200 μL of reaction buffer (100 mmol/L Tris, 2 mmol/L CaCl_2_, 1 mmol/L phenylacetate). The rate of phenylacetate hydrolysis was monitored by the increase in absorbance at the wavelength of 270 nm. CEC was measured using J774.2 macrophages (Sigma-Aldrich, Darmstadt, Germany) cultured in DMEM medium (Dulbecco’s Modified Eagle’s Medium; Sigma-Aldrich, Darmstadt, Germany) containing 10% fetal bovine serum and 1% penicillin/streptomycin, as described [61,62]. Briefly, after seeding on 48-well plates and incubation, macrophages were loaded with ^3^ H-cholesterol (0.5 μCi/mL) in medium (20% FBS, 1% P/S, 0.3 mmol/L 8-(4-chlorophenylthio)-cyclic AMP) overnight. Following rinsing and equilibration in a serum-free medium, macrophages were incubated with the 2.8% apoB-depleted serum at 37 °C for 3 h. CEC was calculated as (radioactivity in the cell culture supernatant)/(radioactivity in supernatant and macrophages).

### 4.5. Brachial Artery Function Assessment

Brachial artery function (FMD and NMD) assessment was performed according to the guidelines given by the International Brachial Artery Reactivity Task Force, the American College of Cardiology [63], and the American Physiological Society [64,65]. The Ultrasound Logiq S8 system (General Electric Medical Systems, Milwaukee, WI, USA) and a 10.0 MHz linear array probe were used for brachial artery imaging. A specially constructed ergonomic pillow and the probe holder were used to reduce involuntary movements of both the subject’s and examiner’s arms. The brachial artery diameter was measured automatically and continuously using the application FloWave.US v. 0.2.0 [66] (Coolbaugh CL, Vanderbilt University Institute of Imaging Science, Nashville, TN, USA) and the program MATLAB R2013a (The MathWorks, Inc.; Natick, MA, USA). The brachial artery was imaged continuously, 2 to 3 cm proximally to the cubital fossa in the longitudinal plane. The brachial artery diameter at rest (basal diameter) was assessed for 2 min. The next step was arterial occlusion using a cuff placed on the forearm (2 cm distally to the cubital fossa) inflated to suprasystolic pressure (50 mmHg above the value of systolic blood pressure) over a period of 5 min. The brachial artery diameter was measured continuously between 30 s prior to and 5 min after the cuff deflation. Having measured the basal brachial artery diameter and the maximum brachial artery diameter after the cuff deflation, FMD was calculated as a percentage of brachial artery dilation ((maximum-basal)/basal). After a 15 min rest, the basal brachial artery diameter was measured again for 2 min. The subject was given 400 μg of glyceryl trinitrate (nitroglycerin) sublingually as an exogenous source of NO to induce endothelial-independent vasodilation. The brachial artery diameter was then again measured continuously for at least 7 min after the drug administration. Comparing the basal brachial artery diameter and the maximum brachial artery diameter after nitroglycerin administration, NMD was calculated as a percentage of brachial artery dilation ((maximum-basal)/basal).

### 4.6. Sample Size Calculations

Sample size calculation was based on the published data regarding serum levels of EL and HDL-C in healthy subjects and those with metabolic syndrome. The sample size for each evaluated variable was calculated using Altman’s nomogram, with α = 0.05 and β = 0.10. The calculation revealed that for observing statistically significant differences at the chosen levels, we needed a total of 22 subjects (11 in each group) for EL and a total of 130 subjects (65 in each group) for HDL-C. Recommendations for minimum sample sizes for FMD were found in the Guidelines for the Ultrasound Assessment of Endothelial-Dependent Flow-Mediated Vasodilation of Brachial Artery [63]. According to these facts, we designed the study with a total of 130 participants, 65 HV and 65 MS patients.

### 4.7. Statistics

Qualitative variables were summarized using absolute and relative frequencies; while quantitative variables were described using mean and standard deviations (SD) or medians and interquartile ranges (q1, q3), depending on the data distribution. To assess differences in the measurements between HV and MS patients, Fisher’s exact test, a t-test, or the Mann–Whitney U test were used, respectively. Correlation analyses using Spearman’s correlation coefficient were performed separately for HV and MS patients. A *p*-value < 0.05 was considered significant. R version 4.1.0 was used for these analyses.

## Figures and Tables

**Table 1:** Differences in demographic and clinical characteristics between HV and MS patients.

Variable	All (N = 130)	HV (N = 65)	MS (N = 65)	*p*
Age (years)	56.0 (50.0, 60.0)	56.0 (50.0, 59.0)	57.0 (50.0, 60.0)	0.440
Sex (female)	62 (47.7%)	31 (47.7%)	31 (47.7%)	1.000
Body weight (kg)	87.5 (75.2, 102.8)	77.0 (68.0, 88.0)	98.0 (86.0, 113.5)	**<0.001**
Body height (m)	1.74 ± 0.10	1.75 ± 0.10	1.73 ± 0.11	0.243
BMI (kg/m^2^)	28.8 (25.1, 32.7)	25.1 (23.7, 28.1)	32.6 (29.8, 35.9)	**<0.001**
Waist circumference (cm)	103.1 ± 16.5	92.2 ± 11.6	113.9 ± 13.2	**<0.001**
Chronic diseases				
Arterial hypertension	60 (46.2%)	0 (0.0%)	60 (92.3%)	**<0.001**
Diabetes mellitus type 2	27 (20.8%)	0 (0.0%)	27 (41.5%)	**<0.001**
Stable angina pectoris	2 (1.5%)	0 (0.0%)	2 (3.1%)	0.496
Atrial fibrillation	2 (1.5%)	0 (0.0%)	2 (3.1%)	0.496
CVI, TIA	1 (0.8%)	0 (0.0%)	1 (1.5%)	1.000
Intermittent claudications	4 (3.1%)	0 (0.0%)	4 (6.2%)	0.119
Deep venous thrombosis	6 (4.6%)	1 (1.5%)	5 (7.7%)	0.208
Pulmonary embolism	2 (1.5%)	0 (0.0%)	2 (3.1%)	0.496
Functions and habits				
Smoking	34 (26.2%)	16 (24.6%)	18 (27.7%)	0.842
Physical activity (≥3 times/week)	105 (80.8%)	58 (89.2%)	47 (72.3%)	**0.025**
Menstrual cycle (female)	18/62 (29.0%)	12/31 (38.7%)	6/31 (19.4%)	0.161

Data are presented as N (%), mean ± standard deviation, or median (q1, q3). Differences between HV and MS patients were tested using Fisher’s exact test, a t-test, or the Mann–Whitney U test, respectively. *p*-values < 0.05 are considered statistically significant and are depicted in bold. BMI, body mass index; cm, centimeter; CVI, cerebrovascular infarction; HV, healthy volunteer; kg, kilogram; m, meter; MS, metabolic syndrome patient; N, number; TIA, transitory ischemic attack.

**Table 2:** Differences in laboratory data between HV and MS patients.

Variable	All (N = 130)	HV (N = 65)	MS (N = 65)	*p*
EL (pg/mL)	353.6 (285.0, 431.2)	345.2 (272.1, 382.9)	367.1 (305.4, 497.0)	**0.002**
Triglycerides (mmol/L)	1.3 (0.9, 1.9)	1.0 (0.8, 1.4)	1.6 (1.1, 2.2)	**<0.001**
Total cholesterol (mmol/L)	5.3 (4.7, 6.1)	5.5 (5.1, 6.0)	5.0 (4.3, 6.2)	0.057
LDL-C (mmol/L)	3.2 (2.5, 3.7)	3.3 (2.8, 3.7)	3.0 (2.3, 3.7)	0.077
HDL-C (mmol/L)	1.4 (1.1, 1.7)	1.6 (1.4, 1.8)	1.2 (1.0, 1.4)	**<0.001**
Glucose (mmol/L)	5.3 (4.9, 5.7)	4.9 (4.8, 5.2)	5.7 (5.3, 6.5)	**<0.001**
Protein (g/L)	73.0 (70.0, 76.0)	72.0 (69.0, 75.0)	75.0 (71.0, 77.0)	**0.002**
Albumin (g/L)	48.0 (46.0, 49.0)	47.0 (46.0, 49.0)	48.0 (45.0, 49.0)	0.465
CRP (µg/mL)	1.8 (0.8, 3.7)	1.2 (0.6, 2.3)	2.4 (1.2, 5.5)	**<0.001**
IL-6 (pg/mL)	3.0 (2.1, 5.3)	2.3 (1.7, 3.0)	4.1 (2.7, 6.8)	**<0.001**
Bilirubin (µmol/L)	8.5 (6.0, 11.6)	9.6 (7.4, 13.3)	7.4 (5.5, 10.4)	**0.012**
AST (U/L)	23.0 (20.0, 27.0)	23.0 (20.0, 25.0)	23.0 (19.0, 32.0)	0.244
ALT (U/L)	24.0 (19.0, 36.0)	22.0 (18.0, 29.0)	30.0 (22.0, 43.0)	**<0.001**
AP (U/L)	61.0 (51.0, 73.0)	60.0 (49.0, 70.0)	65.0 (52.0, 81.0)	0.065
GGT (U/L)	24.5 (15.2, 38.0)	16.0 (13.0, 30.0)	31.0 (21.0, 44.0)	**<0.001**
CK (U/L)	124.5 (83.0, 186.8)	115.0 (81.0, 153.0)	133.0 (86.0, 226.0)	**0.048**
LDH (U/L)	172.0 (150.5, 192.0)	168.0 (147.0, 191.0)	176.0 (158.0, 193.0)	0.365
Urea (mmol/L)	5.3 (4.5, 6.3)	5.0 (4.2, 6.0)	5.6 (4.8, 6.5)	**0.004**
Urate (µmol/L)	297.5 (249.9, 345.1)	273.7 (232.0, 327.2)	315.3 (279.7, 362.9)	**<0.001**
Creatinine (µmol/L)	77.9 (67.3, 87.6)	77.9 (69.0, 89.4)	76.6 (65.5, 87.0)	0.414
eGFR (mL/min/1.73 m^2^)	88.0 (78.0, 97.1)	87.5 (77.2, 93.6)	88.9 (79.1, 98.0)	0.358
Sodium (mmol/L)	139.0 (138.0, 141.0)	140.0 (138.0, 141.0)	139.0 (138.0, 140.0)	**0.041**
Potassium (mmol/L)	4.2 (4.1, 4.6)	4.3 (4.1, 4.5)	4.2 (4.1, 4.6)	0.703
Chloride (mmol/L)	100.0 (98.2, 102.8)	101.0 (99.0, 103.0)	100.0 (98.0, 101.0)	**0.006**

Data are presented as median (q1, q3). Differences between HV and MS patients were tested using the Mann–Whitney U test. *p*-values <0.05 are considered statistically significant and are depicted in bold. LDL-C and eGFR data were available for 60 and 64 MS patients, respectively. ALT, alanine aminotransferase; AP, alkaline phosphatase; AST, aspartate aminotransferase; CK, creatine kinase; CRP, C-reactive protein; eGFR, estimated glomerular filtration rate; EL, endothelial lipase; g, gram; GGT, gamma-glutamyl transpeptidase; HV, healthy volunteer; HDL-C, high-density lipoprotein cholesterol; IL-6, interleukin 6; L, liter; LDH, lactate dehydrogenase; LDL-C, low-density lipoprotein cholesterol; m, meter; µg, microgram; min, minute; mL, milliliter; µmol, micromole; mmol, millimole; MS, metabolic syndrome patient; N, number; pg, picogram; U, unit.

**Table 3:** Differences in serum levels of HDL subclasses between HV and MS patients.

Variable (mg/dL)	All (N = 130)	HV (N = 65)	MS (N = 65)	*p*
HDL-C	58.6 (51.3, 69.2)	65.2 (57.7, 74.5)	52.7 (47.9, 60.6)	**<0.001**
HDL1-C	17.2 (13.8, 22.5)	18.4 (15.1, 26.8)	15.7 (12.9, 20.1)	**0.001**
HDL2-C	8.6 (7.5, 10.2)	9.5 (8.3, 12.6)	8.2 (7.3, 9.7)	**0.001**
HDL3-C	11.2 (10.0, 13.2)	12.2 (10.7, 13.6)	10.4 (9.6, 11.9)	**<0.001**
HDL4-C	20.5 (17.2, 23.9)	22.3 (18.2, 24.7)	19.0 (16.0, 22.7)	**0.001**
HDL-TG	10.5 (9.0, 13.3)	9.9 (8.7, 11.8)	11.4 (9.7, 13.6)	**0.006**
HDL1-TG	3.3 (2.6, 4.4)	3.0 (2.5, 4.2)	3.6 (2.7, 4.6)	0.326
HDL2-TG	1.8 (1.5, 2.3)	1.6 (1.3, 2.1)	2.0 (1.6, 2.5)	**0.001**
HDL3-TG	2.3 (1.9, 2.8)	2.1 (1.7, 2.5)	2.7 (2.1, 3.1)	**<0.001**
HDL4-TG	3.6 (3.0, 4.3)	3.4 (2.5, 3.9)	3.7 (3.3, 4.7)	**<0.001**
HDL-PL	81.9 (72.1, 93.2)	89.4 (79.2, 99.8)	77.1 (67.5, 84.5)	**<0.001**
HDL1-PL	20.5 (16.8, 26.5)	22.1 (18.6, 33.9)	18.9 (14.8, 22.9)	**0.002**
HDL2-PL	13.8 (11.7, 15.9)	14.4 (12.6, 18.6)	13.2 (10.9, 15.4)	**0.017**
HDL3-PL	18.2 (15.9, 20.6)	19.1 (17.4, 20.9)	17.1 (15.2, 19.6)	**0.004**
HDL4-PL	28.8 (25.4, 31.8)	29.9 (26.4, 32.7)	26.3 (23.1, 30.7)	**0.003**
HDL-apoA-I	159.3 (144.5, 178.2)	167.8 (155.7, 183.7)	149.2 (138.1, 166.2)	**<0.001**
HDL1-apoA-I	26.3 (20.0, 34.7)	27.5 (22.3, 45.4)	24.9 (18.5, 30.1)	**0.007**
HDL2-apoA-I	18.7 (16.3, 22.1)	19.6 (17.2, 23.3)	17.5 (15.2, 20.6)	**0.004**
HDL3-apoA-I	30.2 (26.7, 33.3)	30.8 (27.5, 33.5)	28.8 (24.9, 32.5)	**0.040**
HDL4-apoA-I	79.5 (70.8, 89.0)	81.9 (72.7, 91.1)	75.5 (68.3, 86.5)	**0.023**
HDL-apoA-II	35.2 (32.2, 38.4)	36.0 (33.6, 38.6)	33.8 (31.5, 37.5)	**0.039**
HDL1-apoA-II	2.4 (1.9, 3.5)	2.5 (2.1, 4.1)	2.1 (1.6, 3.0)	**0.006**
HDL2-apoA-II	3.9 (3.2, 4.6)	4.0 (3.5, 4.6)	3.7 (3.0, 4.5)	0.111
HDL3-apoA-II	7.3 (6.5, 8.2)	7.3 (6.9, 8.1)	7.1 (6.3, 8.4)	0.739
HDL4-apoA-II	19.8 (17.5, 22.6)	20.9 (18.4, 23.2)	19.2 (17.0, 21.9)	0.060

Data are presented as median (q1, q3). Differences between HV and MS patients were tested using the Mann–Whitney U test. *p*-values < 0.05 are considered statistically significant and are depicted in bold. ApoA-I, apolipoprotein A-I; apoA-II, apolipoprotein A-II; C, cholesterol; dL, deciliter; HV, healthy volunteer; HDL, high-density lipoprotein; mg, miligram; MS, metabolic syndrome patient; N, number; PL, phospholipid; TG, triglyceride.

**Table 4:** Differences in ratios indicating lipid content of HDL particles between HV and MS patients.

Variable	All (N = 130)	HV (N = 65)	MS (N = 65)	*p*
HDL-C / HDL-apoA-I	0.37 (0.35, 0.40)	0.39 (0.36, 0.41)	0.36 (0.33, 0.38)	**<0.001**
HDL1-C / HDL1-apoA-I	0.65 (0.60, 0.71)	0.64 (0.60, 0.69)	0.65 (0.60, 0.72)	0.869
HDL2-C / HDL2-apoA-I	0.48 (0.45, 0.51)	0.49 (0.45, 0.53)	0.47 (0.43, 0.50)	**0.011**
HDL3-C / HDL3-apoA-I	0.39 (0.37, 0.40)	0.40 (0.38, 0.41)	0.38 (0.36, 0.39)	**<0.001**
HDL4-C / HDL4-apoA-I	0.26 (0.24, 0.27)	0.26 (0.25, 0.28)	0.24 (0.23, 0.26)	**<0.001**
HDL-TG / HDL-apoA-I	0.06 (0.06, 0.08)	0.06 (0.05, 0.07)	0.08 (0.06, 0.10)	**<0.001**
HDL1-TG / HDL1-apoA-I	0.12 (0.09, 0.17)	0.11 (0.08, 0.13)	0.15 (0.11, 0.19)	**<0.001**
HDL2-TG / HDL2-apoA-I	0.10 (0.08, 0.13)	0.08 (0.07, 0.11)	0.12 (0.09, 0.16)	**<0.001**
HDL3-TG / HDL3-apoA-I	0.08 (0.06, 0.10)	0.07 (0.06, 0.08)	0.09 (0.07, 0.12)	**<0.001**
HDL4-TG / HDL4-apoA-I	0.04 (0.04, 0.05)	0.04 (0.03, 0.05)	0.05 (0.04, 0.06)	**<0.001**
HDL-PL / HDL-apoA-I	0.52 (0.49, 0.54)	0.53 (0.50, 0.55)	0.51 (0.47, 0.54)	**0.002**
HDL1-PL / HDL1-apoA-I	0.78 (0.74, 0.83)	0.76 (0.73, 0.82)	0.78 (0.74, 0.83)	0.460
HDL2-PL / HDL2-apoA-I	0.74 (0.68, 0.78)	0.73 (0.68, 0.79)	0.74 (0.68, 0.76)	0.694
HDL3-PL / HDL3-apoA-I	0.61 (0.60, 0.63)	0.62 (0.60, 0.63)	0.61 (0.59, 0.63)	0.145
HDL4-PL / HDL4-apoA-I	0.36 (0.34, 0.37)	0.36 (0.35, 0.37)	0.35 (0.33, 0.37)	**0.001**

Data are presented as median (q1, q3). Differences between HV and MS patients were tested with the Mann–Whitney U test. *p*-values < 0.05 are considered statistically significant and are depicted in bold. ApoA-I, apolipoprotein A-I; C, cholesterol; HV, healthy volunteer; HDL, high-density lipoprotein; MS, metabolic syndrome patient; N, number; PL, phospholipid; TG, triglyceride.

**Table 5:** Differences in metrics of HDL function between HV and MS patients.

Variable	All (N = 129)	HV (N = 65)	MS (N = 64)	*p*
AE activity	125.3 (104.5, 145.4)	130.3 (107.5, 151.9)	120.0 (102.1, 135.5)	**0.030**
AE activity / HDL-apoA-I	0.76 (0.65, 0.90)	0.74 (0.65, 0.88)	0.77 (0.64, 0.90)	0.614
CEC	18.3 (17.1, 19.9)	18.8 (17.8, 20.3)	17.5 (16.1, 19.5)	**<0.001**
CEC / HDL-apoA-I	0.11 (0.11, 0.12)	0.11 (0.11, 0.12)	0.11 (0.11, 0.12)	0.277

Data are presented as median (q1, q3). Differences between HV and MS patients were tested using the Mann–Whitney U test. AE activity is presented in mmol/min/mL, CEC in %, and HDL-apoA-I in mg/dL. *p*-values <0.05 are considered statistically significant and are depicted in bold. AE, arylesterase activity of HDL-associated paraoxonase 1; apoA-I, apolipoprotein A-I; CEC, cholesterol efflux capacity of apolipoprotein B-depleted serum; HV, healthy volunteer; HDL, high-density lipoprotein; min, minute; mL, milliliter; mmol, millimole; MS, metabolic syndrome patient; N, number; %, percent.

## Data Availability

Data are available within the article and Supplementary Materials.

## Supplementary Materials

The following supporting information can be downloaded at https://www.mdpi.com/article/10.3390/ijms24032073/s1.

## Abbreviations

AE	arylesterase
Apo A-I	apolipoprotein A-I
Apo A-II	apolipoprotein A-II
CEC	cholesterol efflux capacity
EL	endothelial lipase
FMD	flow-mediated dilation
HDL	high-density lipoprotein
HV	healthy volunteer
MS	metabolic syndrome
NMD	nitroglycerin-mediated dilation
NMR	nuclear magnetic resonance
PL	phospholipid
TG	triglyceride
