# Supplementary material for: Associations between Endothelial Lipase, High-Density Lipoprotein, and Endothelial Function Differ in Healthy Volunteers and Metabolic Syndrome Patients
Source: Int J Mol Sci. 2023 Jan 20;24(3):2073. doi: 10.3390/ijms24032073 (PMC9916974; doi:10.3390/ijms24032073)
Supplement: Supplementary file 1 [file ijms-24-02073-s001.zip › Table S1.pdf]

Table S1. Differences regarding ultrasonographic characteristics of the brachial arteries between HV and MS patients.

Variable	All (N=130)	HV (N=65)	MS (N=65)	p
**Basal measurements 1**				
Systolic blood pressure (mmHg)	130.0 (120.0, 140.0)	120.0 (115.0, 130.0)	140.0 (130.0, 145.0)	**<0.001**
Diastolic blood pressure (mmHg)	80.0 (70.0, 80.0)	70.0 (70.0, 80.0)	80.0 (80.0, 80.0)	**<0.001**
BA basal diameter 1 (mm)	4.39 ± 0.75	4.24 ± 0.66	4.54 ± 0.81	**0.024**
**BA compression**				
Compression pressure (mmHg)	180.0 (170.0, 190.0)	170.0 (165.0, 180.0)	190.0 (180.0, 195.0)	**<0.001**
BA compression diameter (mm)	4.53 ± 0.78	4.38 ± 0.66	4.69 ± 0.86	**0.024**
**After compression**				
BA time to dilation (s)	59.0 (47.0, 86.2)	50.0 (41.0, 67.0)	75.0 (56.0, 99.0)	**<0.001**
BA maximal diameter (mm)	4.71 ± 0.77	4.58 ± 0.67	4.83 ± 0.84	0.055
Δ BA diameter (mm)	0.32 ± 0.14	0.34 ± 0.14	0.30 ± 0.14	0.105
FMD (%)	7.4 ± 3.3	8.1 ± 3.5	6.7 ± 3.1	**0.013**
**Basal measurements 2**				
BA basal diameter 2 (mm)	4.40 ± 0.76	4.25 ± 0.68	4.53 ± 0.81	**0.042**
**After nitroglycerin application**				
BA time to dilation (s)	345.5 (305.8, 402.5)	333.0 (299.0, 397.0)	352.0 (324.0, 426.0)	0.152
BA maximal diameter (mm)	5.14 ± 0.81	5.02 ± 0.71	5.24 ± 0.87	0.124
Δ BA diameter (mm)	0.73 ± 0.23	0.76 ± 0.23	0.71 ± 0.23	0.190
NMD (%)	17.2 ± 6.2	18.4 ± 6.6	16.0 ± 5.7	**0.033**

Data are presented as mean ± standard deviation or median (q1, q3). Differences between HV and MS patients were tested using the t test or Mann-Whitney U test, respectively. P-values <0.05 are considered statistically significant and are depicted in bold. BA compression diameter was measured in 64 HV. Basal measurements 2 as well as all measurements after nitroglycerin application were measured in 57 HV. BA, brachial artey; Δ, change in diameter; FMD, flow-mediated dilation; HV, healthy volunteer; mm, millimeter; mmHg, millimeter of mercury; MS, metabolic syndrome patient; N, number; NMD, nitroglycerin-mediated dilation; s, second; %, percent.
