# Supplementary material for: Associations between Endothelial Lipase, High-Density Lipoprotein, and Endothelial Function Differ in Healthy Volunteers and Metabolic Syndrome Patients
Source: Int J Mol Sci. 2023 Jan 20;24(3):2073. doi: 10.3390/ijms24032073 (PMC9916974; doi:10.3390/ijms24032073)
Supplement: Supplementary file 1 [file ijms-24-02073-s001.zip › Table S2.pdf]

Table S2. Correlation analyses of serum levels of HDL subclasses with FMD and NMD in HV and MS patients.

	**FMD (%)**				**NMD (%)**			
	**HV (N=65)**		**MS (N=65)**		**HV (N=57)**		**MS (N=65)**	
**Variable** (mg/dL)	**r**	**p**	**r**	**p**	**r**	**p**	**r**	**p**
HDL-C	0.14	0.276	0.03	0.810	0.07	0.605	-0.02	0.854
HDL1-C	0.14	0.266	-0.03	0.827	0.11	0.436	-0.08	0.509
HDL2-C	0.09	0.454	-0.06	0.626	0.09	0.512	-0.05	0.697
HDL3-C	0.04	0.723	-0.05	0.693	0.07	0.610	-0.01	0.922
HDL4-C	-0.03	0.804	0.01	0.910	-0.12	0.354	-0.05	0.683
HDL-TG	0.11	0.366	-0.02	0.869	0.20	0.131	-0.09	0.495
HDL1-TG	**0.27**	**0.033**	-0.02	0.849	**0.28**	**0.038**	-0.08	0.505
HDL2-TG	0.12	0.328	-0.01	0.947	0.23	0.085	-0.05	0.721
HDL3-TG	0.03	0.788	-0.02	0.864	0.20	0.146	-0.04	0.726
HDL4-TG	-0.04	0.781	-0.05	0.713	0.02	0.854	-0.14	0.269
HDL-PL	0.14	0.254	-0.03	0.784	0.13	0.347	0.01	0.929
HDL1-PL	0.15	0.237	-0.05	0.672	0.15	0.262	-0.03	0.837
HDL2-PL	0.16	0.215	-0.07	0.554	0.13	0.333	0.00	1.000
HDL3-PL	0.10	0.429	-0.06	0.611	0.11	0.405	0.02	0.879
HDL4-PL	-0.06	0.632	0.04	0.772	-0.12	0.383	0.02	0.857
HDL-apoA-I	0.16	0.196	-0.04	0.763	0.12	0.393	-0.06	0.610
HDL1-apoA-I	0.19	0.129	0.00	0.988	0.16	0.243	0.01	0.930
HDL2-apoA-I	0.18	0.146	-0.07	0.575	0.12	0.366	0.00	0.975
HDL3-apoA-I	0.10	0.436	-0.10	0.412	0.14	0.316	-0.03	0.793
HDL4-apoA-I	-0.05	0.699	0.04	0.734	-0.12	0.368	-0.06	0.652
HDL-apoA-II	-0.01	0.919	-0.06	0.656	0.07	0.625	-0.11	0.376
HDL1-apoA-II	0.14	0.253	-0.01	0.951	0.20	0.140	0.02	0.867
HDL2-apoA-II	0.17	0.173	-0.01	0.941	0.20	0.129	0.03	0.805
HDL3-apoA-II	-0.04	0.732	-0.03	0.786	0.19	0.146	-0.02	0.881
HDL4-apoA-II	-0.11	0.382	-0.02	0.852	-0.13	0.318	-0.11	0.370

Spearman correlation analyses were used to evaluate associations between the serum levels of the HDL subclasses and the metrics of the vessel function. P-values <0.05 are considered statistically significant and are depicted in bold. ApoA-I, apolipoprotein A-I; apoA-II, apolipoprotein A-II; C, cholesterol; dL, deciliter; FMD, flow-mediated dilation; HV, healthy volunteer; HDL, high-density lipoprotein; mg, milligram; MS, metabolic syndrome patient; N, number; NMD, nitroglycerin-mediated dilation; PL, phospholipid; r, Spearman's correlation coefficient; TG, triglyceride; %, percent.
