# Supplementary material for: Associations between Endothelial Lipase, High-Density Lipoprotein, and Endothelial Function Differ in Healthy Volunteers and Metabolic Syndrome Patients
Source: Int J Mol Sci. 2023 Jan 20;24(3):2073. doi: 10.3390/ijms24032073 (PMC9916974; doi:10.3390/ijms24032073)
Supplement: Supplementary file 1 [file ijms-24-02073-s001.zip › Table S3.pdf]

Table S3. Correlation analyses of ratios indicating lipid content of HDL particles with FMD and NMD in HV and MS patients.

	**FMD (%)**				**NMD (%)**			
	**HV (N=65)**		**MS (N=65)**		**HV (N=57)**		**MS (N=65)**	
**Variable**	**r**	**p**	**r**	**p**	**r**	**p**	**r**	**p**
HDL-C / HDL-apoA-I	0.05	0.708	0.05	0.672	-0.01	0.937	0.06	0.622
HDL1-C / HDL1- apoA-I	**-0.30**	**0.015**	-0.10	0.409	-0.16	0.220	-0.15	0.228
HDL2-C / HDL2-apoA-I	0.01	0.949	-0.03	0.838	0.01	0.940	-0.17	0.187
HDL3-C / HDL3-apoA-I	-0.12	0.350	0.10	0.412	-0.11	0.419	-0.03	0.831
HDL4-C / HDL4-apoA-I	-0.04	0.781	-0.05	0.708	-0.09	0.510	-0.01	0.926
HDL-TG / HDL-apoA-I	0.02	0.844	-0.03	0.801	0.08	0.536	-0.07	0.559
HDL1-TG / HDL1-apoA-I	0.03	0.833	-0.01	0.923	0.06	0.637	-0.10	0.413
HDL2-TG / HDL2-apoA-I	0.01	0.966	-0.03	0.815	0.09	0.500	-0.10	0.417
HDL3-TG / HDL3-apoA-I	-0.02	0.896	-0.04	0.731	0.08	0.565	-0.05	0.674
HDL4-TG / HDL4-apoA-I	-0.07	0.600	-0.05	0.709	0.07	0.595	-0.05	0.707
HDL-PL / HDL-apoA-I	0.08	0.519	0.00	0.988	0.10	0.457	0.11	0.403
HDL1-PL / HDL1-apoA-I	**-0.26**	**0.037**	-0.09	0.452	-0.12	0.362	-0.02	0.881
HDL2-PL / HDL2-apoA-I	0.05	0.711	-0.02	0.861	0.11	0.422	0.01	0.937
HDL3-PL / HDL3-apoA-I	-0.06	0.652	0.05	0.690	0.01	0.952	0.17	0.174
HDL4-PL / HDL4-apoA-I	-0.02	0.905	0.07	0.595	0.06	0.640	0.15	0.246

Spearman correlation analyses were used to evaluate associations between the ratios indicating lipid content of HDL particles and the metrics of the vessel function. P-values <0.05 are considered statistically significant and are depicted in bold. ApoA-I, apolipoprotein A-I; C, cholesterol; FMD, flow-mediated dilation; HV, healthy volunteer; HDL, high-density lipoprotein; MS, metabolic syndrome patient; N, number; NMD, nitroglycerin-mediated dilation; PL, phospholipid; r, Spearman's correlation coefficient; TG, triglyceride; %, percent.
