# Supplementary material for: Associations between Endothelial Lipase, High-Density Lipoprotein, and Endothelial Function Differ in Healthy Volunteers and Metabolic Syndrome Patients
Source: Int J Mol Sci. 2023 Jan 20;24(3):2073. doi: 10.3390/ijms24032073 (PMC9916974; doi:10.3390/ijms24032073)
Supplement: Supplementary file 1 [file ijms-24-02073-s001.zip › Table S4.pdf]

Table S4. Correlation analyses of the metrics of the HDL function with FMD and NMD in HV and MS patients.

	**FMD (%)**				**NMD (%)**			
	**HV (N=65)**		**MS (N=64)**		**HV (N=57)**		**MS (N=64)**	
**Variable**	**r**	**p**	**r**	**p**	**r**	**p**	**r**	**p**
AE (mmol/min/mL)	0.15	0.240	-0.02	0.901	0.07	0.601	0.07	0.594
AE / HDL-apoA-I	0.02	0.898	-0.02	0.903	-0.04	0.749	0.09	0.502
CEC (%)	0.04	0.744	-0.02	0.895	0.13	0.342	-0.13	0.324
CEC (%) / HDL-apoA-I	-0.15	0.234	-0.08	0.544	0.04	0.753	-0.11	0.374

Spearman correlation analyses were used to evaluate associations of the metrics of HDL function with the metrics of the vessel function. P-values <0.05 are considered statistically significant and are depicted in bold. AE, arylesterase activity of HDL-associated paraoxonase 1; apoA-I, apolipoprotein A-I; CEC, cholesterol efflux capacity of apolipoprotein B-depleted serum; FMD, flow-mediated dilation; HV, healthy volunteer; HDL, high-density lipoprotein; min, minute; mL, milliliter; mmol, millimole; MS, metabolic syndrome patient; N, number; NMD, nitroglycerin-mediated dilation; r, Spearman's correlation coefficient; %, percent.
