# Supplementary material for: Associations between Endothelial Lipase, High-Density Lipoprotein, and Endothelial Function Differ in Healthy Volunteers and Metabolic Syndrome Patients
Source: Int J Mol Sci. 2023 Jan 20;24(3):2073. doi: 10.3390/ijms24032073 (PMC9916974; doi:10.3390/ijms24032073)
Supplement: Supplementary file 1 [file ijms-24-02073-s001.zip › Table S5.pdf]

Table S5. Correlation analyses of serum levels of EL with serum levels of HDL subclasses in HV and MS patients.

	EL (pg/mL)			
	HV (N=65)		MS (N=65)	
**Variable (mg/dL)**	**r**	**p**	**r**	**p**
HDL-C	0.00	0.994	0.11	0.402
HDL1-C	-0.04	0.723	0.23	0.060
HDL2-C	-0.12	0.351	0.02	0.856
HDL3-C	-0.13	0.317	-0.12	0.346
HDL4-C	0.19	0.130	-0.01	0.923
HDL-TG	0.05	0.682	-0.06	0.639
HDL1-TG	-0.06	0.637	0.08	0.520
HDL2-TG	-0.04	0.738	-0.02	0.894
HDL3-TG	0.01	0.914	-0.09	0.456
HDL4-TG	0.13	0.297	-0.16	0.203
HDL-PL	-0.10	0.420	0.01	0.936
HDL1-PL	-0.09	0.454	0.17	0.188
HDL2-PL	-0.19	0.128	-0.03	0.831
HDL3-PL	-0.16	0.208	-0.07	0.565
HDL4-PL	0.10	0.421	-0.08	0.515
HDL-apoA-I	-0.01	0.935	-0.03	0.820
HDL1-apoA-I	-0.08	0.510	0.17	0.185
HDL2-apoA-I	-0.12	0.341	-0.03	0.788
HDL3-apoA-I	-0.11	0.379	-0.18	0.152
HDL4-apoA-I	0.20	0.107	-0.06	0.623
HDL-apoA-II	0.05	0.665	-0.20	0.107
HDL1-apoA-II	-0.19	0.138	-0.13	0.312
HDL2-apoA-II	-0.20	0.116	-0.15	0.229
HDL3-apoA-II	-0.10	0.445	**-0.25**	**0.047**
HDL4-apoA-II	0.20	0.107	-0.12	0.344

Spearman correlation analyses were used to evaluate associations between the serum levels of EL and the serum levels of HDL subclasses. P-values <0.05 are considered statistically significant and are depicted in bold. ApoA-I, apolipoprotein A-I; apoA-II, apolipoprotein A-II; C, cholesterol; dL, deciliter; EL, endothelial lipase; HV, healthy volunteer; HDL, high-density lipoprotein; mg, milligram; mL, milliliter; MS, metabolic syndrome patient; N, number; pg, picogram; PL, phospholipid; r, Spearman's correlation coefficient; TG, triglyceride.
