# Supplementary material for: Associations between Endothelial Lipase, High-Density Lipoprotein, and Endothelial Function Differ in Healthy Volunteers and Metabolic Syndrome Patients
Source: Int J Mol Sci. 2023 Jan 20;24(3):2073. doi: 10.3390/ijms24032073 (PMC9916974; doi:10.3390/ijms24032073)
Supplement: Supplementary file 1 [file ijms-24-02073-s001.zip › Table S6.pdf]

Table S6. Correlation analyses of serum levels of EL with ratios indicating lipid content of HDL particles in HV and MS patients.

	EL (pg/mL)			
	HV (N=65)		MS (N=65)	
**Variable**	**r**	**p**	**r**	**p**
HDL-C / HDL-apoA-I	-0.01	0.907	0.23	0.071
HDL1-C / HDL1-apoA-I	0.21	0.088	0.04	0.727
HDL2-C / HDL2-apoA-I	-0.07	0.599	0.06	0.613
HDL3-C / HDL3-apoA-I	-0.02	0.890	0.03	0.799
HDL4-C / HDL4-apoA-I	0.09	0.488	0.02	0.846
HDL-TG / HDL-apoA-I	-0.02	0.890	-0.02	0.871
HDL1-TG / HDL1-apoA-I	0.03	0.789	0.03	0.830
HDL2-TG / HDL2-apoA-I	-0.02	0.885	0.03	0.783
HDL3-TG / HDL3-apoA-I	0.01	0.938	-0.01	0.943
HDL4-TG / HDL4-apoA-I	0.04	0.759	-0.08	0.501
HDL-PL / HDL-apoA-I	**-0.25**	**0.041**	0.07	0.581
HDL1-PL / HDL1-apoA-I	-0.02	0.904	0.00	0.977
HDL2-PL / HDL2-apoA-I	-0.24	0.052	0.04	0.775
HDL3-PL / HDL3-apoA-I	-0.17	0.176	0.00	0.974
HDL4-PL / HDL4-apoA-I	-0.18	0.141	-0.08	0.524

Spearman correlation analyses were used to evaluate associations between the serum levels of EL and the ratios indicating lipid content of HDL particles. P-values <0.05 are considered statistically significant and are depicted in bold. ApoA-I, apolipoprotein A-I; apoA-II, apolipoprotein A-II; C, cholesterol; EL, endothelial lipase; HV, healthy volunteer; HDL, high-density lipoprotein; mL, milliliter; MS, metabolic syndrome patient; N, number; pg, picogram; PL, phospholipid; r, Spearman's correlation coefficient; TG, triglyceride.
