# Supplementary material for: Associations between Endothelial Lipase, High-Density Lipoprotein, and Endothelial Function Differ in Healthy Volunteers and Metabolic Syndrome Patients
Source: Int J Mol Sci. 2023 Jan 20;24(3):2073. doi: 10.3390/ijms24032073 (PMC9916974; doi:10.3390/ijms24032073)
Supplement: Supplementary file 1 [file ijms-24-02073-s001.zip › Table S7.pdf]

Table S7. Correlation analyses of serum levels of EL with the metrics of the HDL function in HV and MS patients.

	EL (pg/mL)			
	HV (N=65)		MS (N=64)	
**Variable**	**r**	**p**	**r**	**p**
AE activity	0.17	0.178	-0.24	0.058
AE activity / HDL-apoA-I	0.15	0.242	-0.22	0.074
CEC	-0.08	0.529	-0.14	0.282
CEC/ HDL-apoA-I	-0.11	0.387	-0.12	0.325

Spearman correlation analyses were used to evaluate associations between the serum levels of EL and the metrics of HDL function. AE activity is presented in mmol/min/mL, HDL-apoA-I in mg/dL, and CEC in %. P-values <0.05 are considered statistically significant. AE, arylesterase activity of HDL-associated paraoxonase 1; apoA-I, apolipoprotein A-I; CEC, cholesterol efflux capacity of HDL; EL, endothelial lipase; HV, healthy volunteer; HDL, high-density lipoprotein; min, minute; mL, milliliter; mmol, millimole; MS, metabolic syndrome patient; N, number; pg, picogram; r, Spearman's correlation coefficient; %, percent.
