# Supplementary material for: Associations between Endothelial Lipase, High-Density Lipoprotein, and Endothelial Function Differ in Healthy Volunteers and Metabolic Syndrome Patients
Source: Int J Mol Sci. 2023 Jan 20;24(3):2073. doi: 10.3390/ijms24032073 (PMC9916974; doi:10.3390/ijms24032073)
Supplement: Supplementary file 1 [file ijms-24-02073-s001.zip › Table S8.pdf]

Table S8. Correlation analyses of serum levels of EL with BMI, IL-6, and CRP in HV and MS patients.

	**EL (pg/mL)**			
	**HV (N=65)**		**MS (N=65)**	
**Variable**	**r**	**p**	**r**	**p**
BMI (kg/m^2)	0.03	0.834	0.42	**< 0.001**
IL-6 (pg/mL)	0.03	0.816	0.38	**0.002**
CRP (µg/mL)	0.10	0.427	0.30	**0.014**

Spearman correlation analyses were used to evaluate associations between the serum levels of EL and BMI, IL-6 and CRP. P-values <0.05 are considered statistically significant and are depicted in bold. BMI, body mass index; CRP, C-reactive protein; EL, endothelial lipase; HV, healthy volunteer; IL-6, interleukine-6; mL, milliliter; µg, microgram; MS, metabolic syndrome patient; N, number; pg, picogram; r, Spearman's correlation coefficient.
